# TMJ Effusion and Anterior Disk Displacement: A Systematic Review and Meta-Analysis

**DOI:** 10.3390/dj14100663

**Published:** 2026-10-12

**Authors:** Marco Portelli, Ignazio Russo, Angela Mirea Bellocchio, Angela Militi, Riccardo Nucera

**Affiliations:** Department of Biomedical, Dental Science and Morphological and Functional Images, University of Messina, 98122 Messina, Italy; mportelli@unime.it (M.P.); angelamirea@live.it (A.M.B.); amiliti@unime.it (A.M.); riccardo.nucera@unime.it (R.N.)

**Keywords:** joint effusion, ADDWR, ADDWOR, inflammatory cytokines

## Abstract

**Background:** The objective of this study was to assess the association between joint effusion and anterior disk displacement. Specifically, it aimed to determine whether joint effusion is more associated with anterior disk displacement without reduction (ADDWOR) compared with displacement with reduction (ADDWR). **Methods:** The literature review for this study was conducted using PubMed, Scopus, SciELO, the Virtual Health Library (including LILACS), and The Cochrane Library (Trials and Reviews). Grey literature was also searched, specifically: ClinicalTrials.gov and the International Clinical Trials Registry Platform (ICTRP Search Portal, World Health Organization). The review was conducted according to the PRISMA 2020 statement, and the search strategy followed the PECO (Population, Exposure, Comparison, Outcome) protocol. Study quality was assessed with the Newcastle–Ottawa Scale (cohort studies) and the Joanna Briggs Institute critical appraisal tools (cross-sectional and case–control studies). All raw event counts were independently re-extracted and cross-checked against the original source tables for every included study, and a design-effect sensitivity analysis was performed to explore the potential impact of the non-independence of bilaterally assessed joints. **Results:** Seventeen studies were included, comprising 4254 temporomandibular joint (TMJ)-level observations (2256 ADDWOR joints, 1998 ADDWR joints) from approximately 2980 patients. The meta-analysis showed a statistically significant association between joint effusion and disc displacement, with a random-effects odds ratio (OR) of 1.70 (95% CI: 1.42–2.03; *p* < 0.0001; I^2^ = 24.1%), consistent with the common-effect model (OR = 1.72; 95% CI: 1.50–1.97; *p* < 0.0001). The pooled estimate was not materially altered by the omission of any single study; Egger’s test did not detect funnel-plot asymmetry (*p* = 0.94), although publication or small-study effects cannot be excluded; and an approximate design-effect sensitivity analysis for bilateral joints, under assumed intraclass correlation coefficients of 0–0.8, gave similar estimates. These results indicate an unadjusted joint-level association: joint effusion was more frequently observed in joints with anterior disc displacement without reduction (ADDWOR) than in joints with displacement with reduction (ADDWR). **Conclusions:** These findings indicate a significant unadjusted joint-level association between joint effusion and disc displacement without reduction. Because in all included studies disc position and effusion were assessed on a single MRI examination, this association should not be interpreted as evidence of causality; further prospective investigation is needed to confirm the association and clarify the underlying mechanisms.

## 1. Introduction

Temporomandibular disorders (TMDs) represent the most frequent orofacial pain of non-dental origin [1] and are a set of symptoms involving either only the temporomandibular joints (TMJ), only the masticatory muscles, or both. TMDs affect between 5 and 12% of the population, with a higher prevalence in females and with an economic reflection that can be evaluated in a yearly cost of USD 4 billion [2]. According to Abdalla-Aslan et al. [3], temporomandibular disorders affect 11% of children/adolescents and 31% of the adult/senile population. One of the most studied aspects in TMD is internal TMJ displacement, defined as an abnormal relationship between the articular disc and the mandibular condyle [1]. The most frequent displacement is anterior displacement, which can be classified into two main categories: with reduction (ADDWR) and without reduction (ADDWOR). Displacement with reduction occurs when the bony heads are restored to their original anatomical position and, according to Abdalla-Aslan et al. [3], is the most frequent form of disorder. Audible clicking, popping, or snapping noises are characteristic features of ADDWR, occurring during disc reduction with condylar movement [4]. In cases of ADDWOR, instead, the disc does not reduce with opening of the mouth; this condition can be associated with a limitation of mouth opening and on magnetic resonance imaging, it is possible to observe an intermediate zone of the disc anterior to the condyle [4]. Joint effusion is a clinical condition characterized by an accumulation of fluid in the joint cavity, often associated with inflammation, pain, and articular movement reduction [5,6]. TMJ synovial fluid is derived from plasma and has several functions, including delivery of nutrients such as lipids, cholesterol, phospholipids, and hyaluronic acid; joint lubrication; and catabolite disposal [7]. The gold standard examination in the evaluation of TMDs (Temporomandibular disorders) is magnetic resonance imaging (MRI), allowing a complete evaluation of soft tissues and joint effusion [8]. Through MRI, it is possible to acquire positional information about the disc [9], quantitative measurements of synovial fluid [10], and an evaluation of the condition of retrodiscal tissues [11] and bone marrow [12]. Most studies conducted with MRI have concentrated on signal alterations in joint compartments, revealing the presence of fluid resulting from the exudation of inflamed retrodiscal tissues and inflammatory changes in the synovial membrane, leading to joint effusion [13]. On T2-weighted MRI images, TMJ effusion commonly appears as an area of increased signal intensity [14] and may reflect a local problem caused by trauma or a systemic disease like rheumatoid or psoriatic arthritis. Furthermore, abnormalities in the morphology and position of the disc have been correlated with TMJ effusion [15], suggesting that effusion might be an inflammatory response to a compromised disc-condyle relationship [16]. It has been suggested by some authors that the relationship between effusion and disc displacement is more evident in the advanced stages of displacement, indicating a chronic evolution of joint disorder [17]. The relationship between joint effusion and anterior disc displacement of the TMJ is widely debated in the scientific literature, with different results. Some studies found that compared to joints with normal disc position, joints with anterior disc displacement with reduction (ADDWR) have a higher probability of joint effusion and degenerative changes [18]. These findings suggest a connection between ADDWR and joint inflammation. Other studies, including those by Thomas et al. and Manfredini et al. [19,20], indicate that joint effusion is more commonly observed in patients with anterior disc displacement without reduction (ADDWOR). However, the mechanisms explaining this association remain unclear and need to be investigated. Finally, some evidence [21] found a significant association between joint effusion and anterior disc displacement without significant differences between ADDWR and ADDWOR. These results reflect the complexity of the pathogenesis of temporomandibular disorders and suggest that the role of joint effusion can be different depending on the type and stage of disc displacement. Through an analysis of available clinical data, the purpose of the present study is to investigate if joint effusion is more associated with ADDWOR compared with ADDWR.

## 2. Materials and Methods

### 2.1. Protocol

This systematic review was conducted following the PRISMA 2020 statement [22] (PRISMA 2020 checklist, File S1) and complies with the recommendations of the Cochrane Handbook for Systematic Reviews of Interventions. This review plan received the ID CRD420250649284 upon its registration in the PROSPERO.

### 2.2. Search Strategy and Sources of Information

The search was conducted in five electronic databases: MEDLINE (via PubMed), Scopus, SciELO, Virtual Health Library (including LILACS database), and The Cochrane Library (Trials and Reviews). Grey literature was also searched, specifically: www.ClinicalTrials.gov (accessed on 14 September 2026) and International Clinical Trials Registry Platform (searched by ICTRP Search Portal—World Health Organization; https://trialsearch.who.int, accessed on 14 September 2026).

### 2.3. Eligibility Criteria

Criteria for inclusion (PECO):

**Participants:**

1. Observational studies exploring the relationship between anterior disc displacement (ADD) and joint effusion.
2. Study population consisting of subjects of both sexes.
3. Inclusion of studies with available data regarding the diagnosis of joint displacement and effusion.

**Exposure:**

Group of subjects with disc displacement without reduction (ADDWOR).

**Comparison:**

Group of subjects with disc displacement with reduction (ADDWR).

**Outcomes:**

Presence and severity of joint effusion, evaluated on magnetic resonance imaging (MRI).

**Studies:**

1. Eligible studies included cross-sectional, cohort, or case–control designs.
2. Only studies with more than 15 TMJs in each group (ADDWR and ADDWOR) were included. This threshold, pre-specified in the protocol registered with PROSPERO (CRD420250649284), reflects a conventional minimum sample size adopted to exclude excessively small, clinically anecdotal case series, while remaining feasible given the limited number of published studies reporting joint-level ADDWOR/ADDWR effusion data.

**Exclusion criteria:**

1. Studies including case series, case reports or cohorts with fewer than 15 TMJs per group.
2. Books and editorials.

Seven sources (five electronic databases and two trial registers) were used to search for studies that met the inclusion criteria:

1. MEDLINE (via PubMed);
2. Cochrane Library;
3. Scielo;
4. Virtual Health Library;
5. Clinical trials government;
6. International Clinical Trials Registry Platform;
7. Scopus.

These databases were last searched on 14 September 2026. During the study selection process, duplicate articles were excluded, ensuring that each study was considered only once in the analysis. The search focused on studies reported in English and was limited to those that included human subjects. The database-specific search strings are reported in Table S5.

### 2.4. Data Extraction

Title and abstract screening, as well as full-text assessment for eligibility, were performed independently by two reviewers (I.R. and A.M.B.) using a pre-designed Excel (Microsoft Corporation, Redmond, WA, USA) extraction sheet with the following elements: authors, year, study design, country, definition of joint effusion, definition of disc displacement, cases, and controls; disagreements were resolved by discussion with a third author (M.P.).

### 2.5. Quality Assessment

Study design was classified as follows: cohort, when the original authors reported a cohort design; case–control, when a separate group of asymptomatic volunteers was enrolled as controls; and analytical cross-sectional, in all other studies, in which disc position and effusion were assessed on the same MRI examination. For the quality assessment of cohort studies (Table S1), the Newcastle–Ottawa Scale (NOS) was used [23], with three domains (selection, comparability and outcome). The quality assessment of cross-sectional (Table S2) and case–control studies (Table S3) was performed using the corresponding Joanna Briggs Institute (JBI) critical appraisal checklists [24] (8 and 10 items, respectively; the items are listed in the table legends). Each domain was rated as low risk, unclear or high risk. A study was judged to be at an overall low risk of bias when no domain was rated high risk and no more than one domain was rated unclear; otherwise, it was judged as having some concerns. No separate overall high-risk category was used: studies with one or more domains rated high risk (in all cases, items related to confounding) were classified as having some concerns, and this was taken into account by interpreting the pooled estimate as an unadjusted association. Two authors independently conducted the assessment, resolving any discrepancies through consultation with a senior investigator.

### 2.6. Statistical Analysis

Data on the diagnosis of joint dislocation and effusion were extracted from the individual studies, and a meta-analysis was conducted using the Mantel–Haenszel method for the common-effect model and the inverse-variance (DerSimonian–Laird) method for the random-effects model, with the odds ratio (OR) as the effect measure. Heterogeneity was assessed using Cochran’s Q test and the I^2^ statistic. A leave-one-out sensitivity analysis was conducted to examine the influence of individual studies on the pooled estimate and on overall heterogeneity. Potential publication/small-study bias was assessed using funnel plot analysis (Figure S1) and Egger’s linear regression test for funnel plot asymmetry. All statistical analyses were performed in R (version 4.2.3; R Foundation for Statistical Computing, Vienna, Austria) using the meta package (version 8.3.0). Pairwise meta-analyses were fitted with the metabin() function (Mantel–Haenszel method for the common-effect model, DerSimonian–Laird for the random-effects model); confidence intervals were computed using the standard normal (Wald-type) approximation on the log-odds-ratio scale, without the Hartung–Knapp–Sidik–Jonkman adjustment (default package settings). The leave-one-out sensitivity analysis used metainf(), and the funnel plot and Egger’s test used funnel() and metabias(), respectively. By default, metabin() applies a continuity correction of 0.5 only to studies with at least one zero cell, which was retained for the present analysis. Because 16 of the 17 included studies reported bilateral MRI findings (i.e., non-independent, clustered joint-level observations from the same patient), an additional design-effect sensitivity analysis was performed, adapting the approximate cluster-adjustment method described in the *Cochrane Handbook for Systematic Reviews of Interventions*, Chapter 23, Section 23.1.4 (“Approximate analyses of cluster-randomized trials for a meta-analysis: effective sample sizes”) [25]. This method was originally developed for cluster-randomized trials; here, the same design-effect logic was applied to the analogous within-patient clustering arising from bilateral TMJ assessment (approximately two joints per patient in 16 of the 17 studies). Following this approach, both the event count and the total count for each study were reduced to their effective sample size by dividing the design effect, DEFF = 1 + (M − 1) × ICC, where M is the average cluster size (M = 2 joints per patient) and ICC is the assumed intraclass correlation coefficient, across a range of assumed values (ICC = 0 to 0.8). Because the true within-patient correlation is unknown, this analysis was regarded as an approximate assessment under assumed correlations. For studies reporting effusion on an ordinal scale, effusion was dichotomised as absent (grade 0) versus present (any higher grade) (Table S6).

## 3. Results

### 3.1. Study Selection

Following a systematic literature search, a total of 2294 articles were found: 1268 titles on PubMed, 7 on Scopus, 552 on Virtual Health Library, 432 on the Cochrane Library, 16 on SciELO, 17 on ClinicalTrials.gov and 2 on the International Clinical Trials Registry Platform. After the removal of duplicates, 1576 papers remained for the screening of titles and abstracts. Subsequently, 27 studies were considered eligible for full-text evaluation.

After full-text reading, 10 studies were excluded (wrong outcome measure, *n* = 7; review article, *n* = 2; ineligible study design, *n* = 1; see Table S4 for details), and 17 studies were finally included for qualitative and quantitative synthesis.

**Figure 1:**
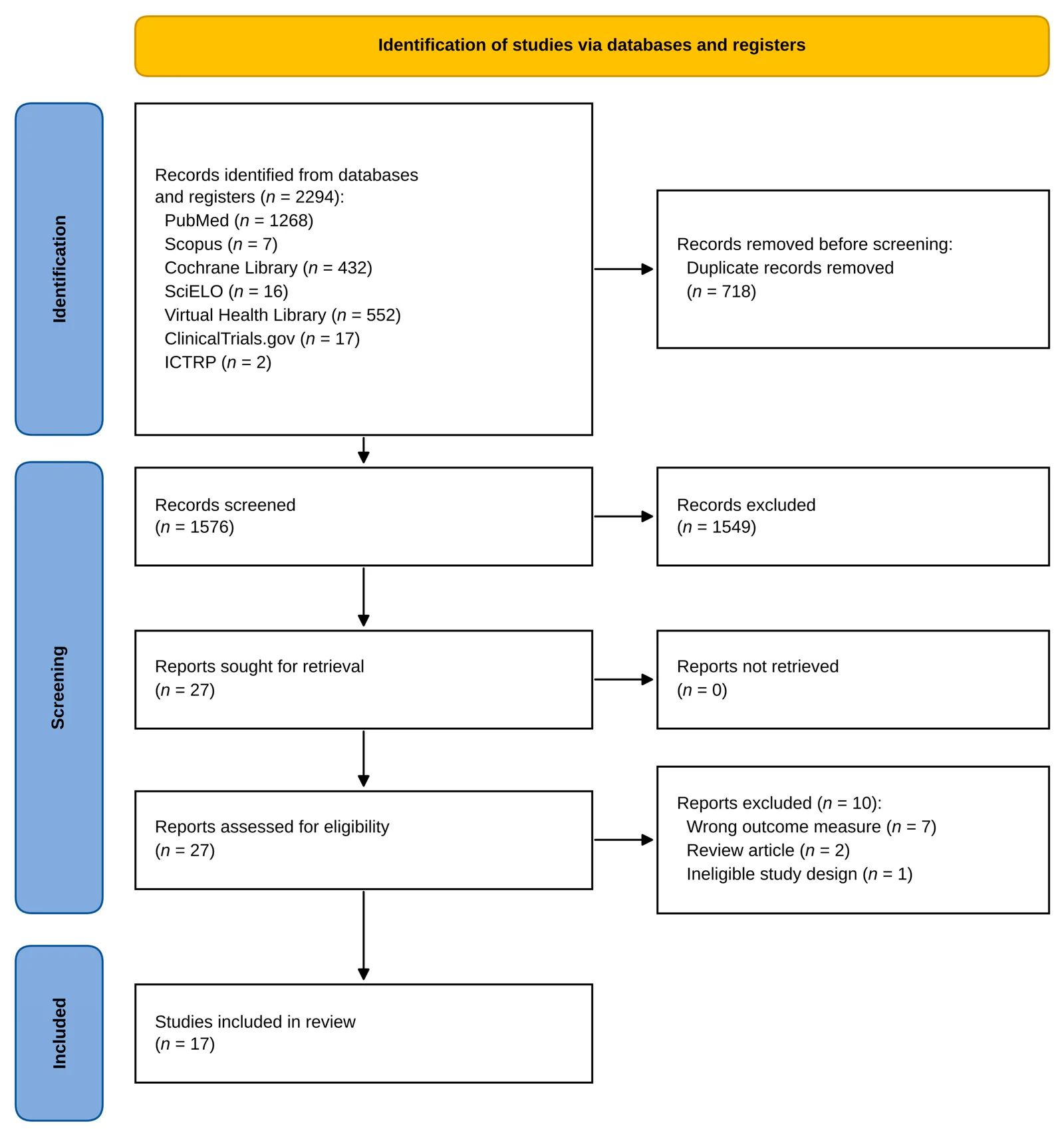
Flow chart of study selection performed according to PRISMA guidelines.

An overview of the selection process is shown in Figure 1.

### 3.2. Characteristics of the Included Studies

The 17 studies enrolled a total of approximately 2980 patients (Table 1), with mean age ranging from approximately 27 [26] to 55.3 [20] years; sample sizes ranged from 41 [21] to 766 participants [27]. Because 16 of the 17 studies assessed TMJs bilaterally (in Liao et al. [26], both joints were included when available), the dataset used for the quantitative synthesis comprises 4254 TMJ-level observations (2256 ADDWOR joints and 1998 ADDWR joints), reflecting the joint, rather than the patient, as the unit of analysis. The aggregate patient count is the sum of the patients enrolled in each study and should be regarded as approximate. It includes the 11 asymptomatic volunteers enrolled in Westesson and Brooks [5], as well as patients in studies in which only a subset of joints met the inclusion criteria [28,29,30]. Mizuhashi et al. 2024 [31] did not report the number of patients separately from the number of joints (97 TMJs); because the reported sex distribution (12 men, 85 women) sums to 97, one joint per patient was assumed. If all joints in that study had instead been bilateral, the patient total would be approximately 2932. The studies were conducted in 7 different countries. Two studies were cohort studies [19,28], thirteen were analytical cross-sectional studies [6,18,20,21,26,27,29,30,31,32,33,34,35] and two were case–control studies [5,36]. Designs were classified according to the criteria reported in Section 2.5; none of the studies classified as cross-sectional reported a cohort design or any follow-up. Whatever the original design, disc position and effusion were assessed on the same MRI examination in every study, so the data contributing to the ADDWOR versus ADDWR comparison are cross-sectional. All studies reviewed shared a similar approach in the use of MRI for the diagnosis of temporomandibular disorders (TMD), focusing on the assessment of joint disc position and joint effusion. Most of the studies utilized MRI machines with a magnetic field strength of 1.5 Tesla, ensuring high image resolution. However, some variations were observed: Xu et al. (2023) [36] used a 3.0 T MRI for enhanced resolution, whereas Hosgor et al. 2019 [6] employed a 0.5 T MRI. Additionally, Khawaja et al. (2016) [28] incorporated CBCT alongside MRI to evaluate hard tissues and bony integrity of the TMJ, focusing on degenerative joint disorders (DJDs), while MRI was used to evaluate soft tissues, such as the presence of joint effusion and joint disc position/shape [28]. Another commonality is the use of various mouth positions (open or closed) to assess dynamic changes in the temporomandibular joint. The most common sequences were T1- and T2-weighted sequences, with particular attention to joint effusion (presence of fluid) and disc morphology (biconcave, biconvex, etc.). Images were acquired with T1- and T2-weighted sequences in four studies [6,20,33,35], with proton-density (PD) and T2-weighted sequences in six [19,29,30,31,34,36], and with T1-weighted, T2-weighted and PD sequences in two [21,27]; one study graded effusion on fat-suppressed T2-weighted images [26], and the remaining studies did not report the sequences in detail [5,18,28,32]. A summary of the descriptive characteristics of the studies can be found in Table 1.

### 3.3. Risk of Bias in Studies

The two cohort studies were assessed with the Newcastle–Ottawa Scale (Table S1). Selection was rated as low risk in both; comparability was unclear in Khawaja 2016 [28] and outcome was unclear in Thomas 2018 [19], and both were judged at overall low risk of bias. The thirteen cross-sectional studies were assessed with the JBI Critical Appraisal Checklist for analytical cross-sectional studies (Table S2). Liao 2025 [26], Mizuhashi 2022 [29], Ogura 2019 [32] and Roh 2012 [18] were judged at overall low risk of bias. Hosgor 2019 [6], Koh 2013 [33], Li 2024 [27], Manfredini 2009 [20], Mizuhashi 2024 [31], Orlando 2009 [35], Rudisch 2001 [21], Yasan 2023 [34] and Yesiltepe 2022 [30] showed some concerns, mainly related to the identification of and adjustment for confounding factors. Koh 2013, Mizuhashi 2024, Orlando 2009 and Rudisch 2001 [21,31,33,35] were rated at high risk for the item “strategies to deal with confounding factors”, and Koh 2013, Manfredini 2009 and Orlando 2009 [20,33,35] also for “confounding factors identified”. The two case–control studies were assessed using the JBI Critical Appraisal Checklist for case–control studies (Table S3). Both studies (Westesson 1992 [5], Xu 2023 [36]) showed some concerns overall, consistently related to inadequate strategies to deal with confounding factors (rated high risk in both). No study was excluded from the synthesis based on quality assessment. Because confounding was rarely addressed in the primary studies, the pooled estimate should be interpreted as an unadjusted joint-level association.

### 3.4. Results of Individual Studies

Several studies have investigated the association between anterior disc displacement of the TMJ and the presence of joint effusion, reporting how the amount of intra-articular synovial fluid differs according to the type of disc displacement. Xu et al. (2023) [36] observed that patients with anterior disc displacement with reduction (ADDWR) and without reduction (ADDWOR) exhibited greater joint effusion compared to subjects with a normal disc position, with type III morphology showing the most significant effusion. Mizuhashi et al. (2024) [31] found that the amount of synovial fluid differed between ADDWR and ADDWOR cases, with grade 2 effusion being more frequent in ADDWR and grade 3 more common in ADDWOR patients (*p* = 0.024), although the presence and region of effusion did not differ significantly between the two groups; consistently, in the joint-level data extracted for the present review the proportion of joints with effusion was similar in the two groups (ADDWOR 44/62; ADDWR 26/35). Yasan et al. (2023) [34] identified an association between ADDWOR and grade 3 joint effusion, indicating that more severe effusion was more frequently observed in joints with ADDWOR. An association between anterior disc displacement and joint effusion, generally relative to joints with a normal disc position, was reported by most of the included studies [5,6,19,20,21,26,27,28,29,30,32,34,35,36], whereas Koh et al. [33] found no statistically significant relationship between the two conditions (*p* > 0.05). Roh et al. (2012) [18] reported that patients with ADDWR had an odds ratio of 2.85 (*p* < 0.001) for joint effusion compared to subjects with a normal disc position, whereas ADDWOR patients showed even higher odds, with an odds ratio of 4.61 (*p* < 0.001). Similarly, Thomas et al. (2018) [19] found a significant association between disc position and joint effusion (*p* < 0.01), with a particularly strong correlation in ADDWOR cases and disc perforation. Mizuhashi et al. (2022) [29] also reported a higher presence of joint effusion in patients with ADDWOR compared to other conditions, indicating that joint effusion was more frequently observed in joints without disc reduction. Li et al. (2024) [27] found that patients with ADDWR had 1.740-fold higher odds of joint effusion compared with those with a normal disc position. Finally, Orlando et al. (2009) [35] identified a significant correlation between disc displacement and joint effusion (*p* < 0.01), with an odds ratio of 3.1 for all types of displacement. In particular, joints with ADDWOR showed the highest proportion of MRI-detected effusion. Similarly, Liao et al. (2025) [26] reported that the grade of joint effusion differed between ADDWR and ADDWOR joints and remained associated with ADDWOR in a multivariable analysis. It should be noted that several of the odds ratios reported above (e.g., Roh et al. [18], Khawaja et al. [28]) were computed by the original authors against a reference category of normal disc positions rather than against ADDWR, and the association reported by Li et al. [27] above was derived from a balanced, outcome-selected sub-sample used by the original authors for multivariable regression. For the quantitative synthesis presented in Section 3.5, raw joint-level 2 × 2 counts were therefore re-extracted directly from each source study’s full-cohort tables (with source table and page recorded for every study) to compute a single, harmonized ADDWOR-vs-ADDWR odds ratio for each study, ensuring a consistent comparison across the pooled analysis.

### 3.5. Meta-Analysis

**Figure 2:**
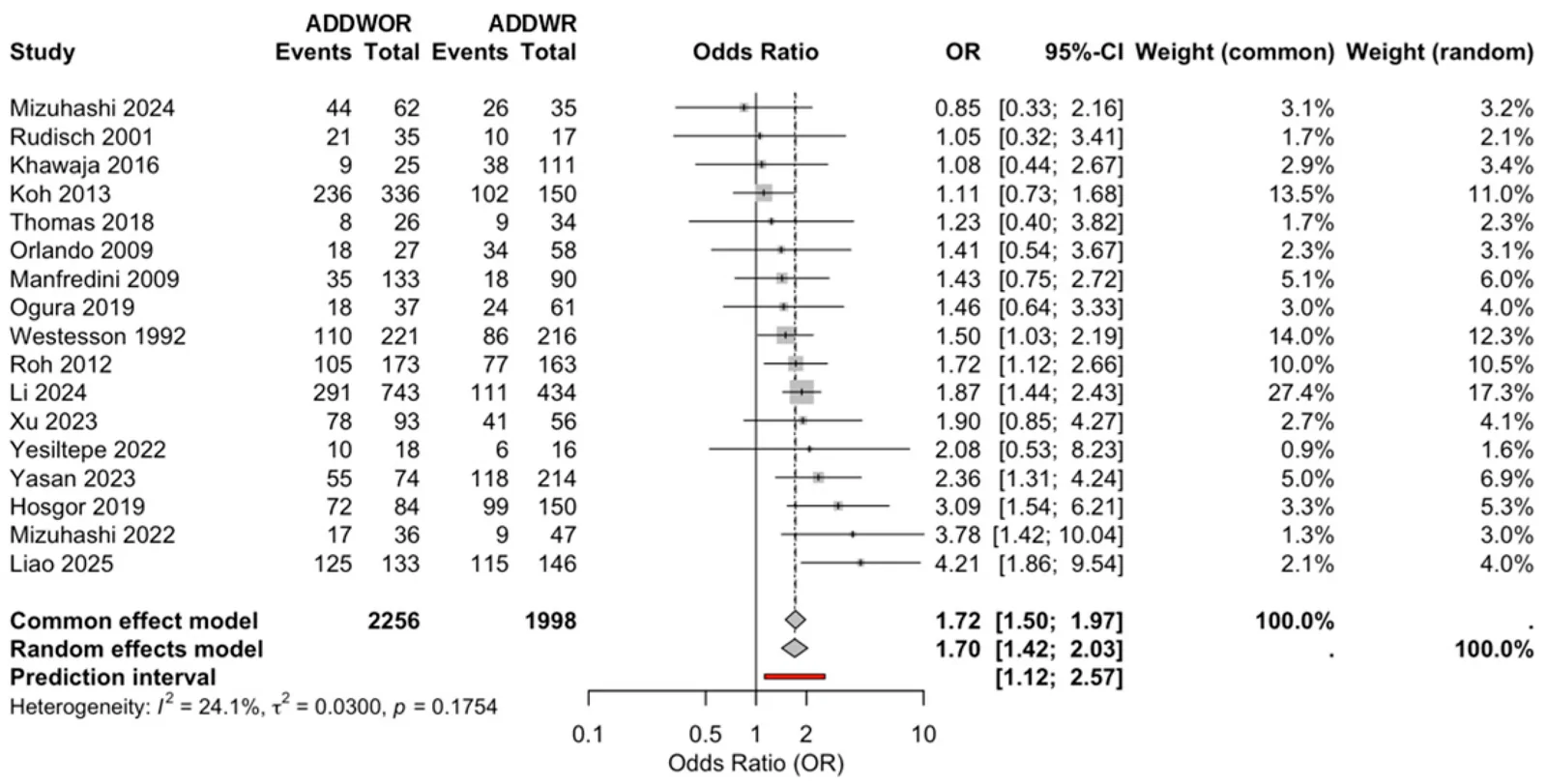
Forest plot for the association between anterior disc displacement without reduction (ADDWOR) and joint effusion, compared with displacement with reduction (ADDWR); k = 17 studies, 4254 TMJ-level observations (common-effect OR = 1.72, 95% CI: 1.50–1.97; random-effects OR = 1.70, 95% CI: 1.42–2.03). Grey squares represent the study-specific ORs (square size proportional to the study weight), and horizontal lines their 95% CIs; diamonds represent the pooled common-effect and random-effects estimates; the red bar represents the 95% prediction interval; the solid vertical line indicates no association (OR = 1), and the dashed vertical line indicates the pooled estimate. Included studies: Mizuhashi 2024 [31]; Rudisch 2001 [21]; Khawaja 2016 [28]; Koh 2013 [33]; Thomas 2018 [19]; Orlando 2009 [35]; Manfredini 2009 [20]; Ogura 2019 [32]; Westesson 1992 [5]; Roh 2012 [18]; Li 2024 [27]; Xu 2023 [36]; Yesiltepe 2022 [30]; Yasan 2023 [34]; Hosgor 2019 [6]; Mizuhashi 2022 [29]; Liao 2025 [26].

A total of 17 studies were included, analyzing the association between joint effusion and anterior disc displacement across 4254 TMJ-level observations (2256 ADDWOR joints, 1998 ADDWR joints; approximately 2980 patients). These studies consisted of two cohort studies, thirteen cross-sectional studies and two case–control studies; in all of them, disc position and effusion were assessed on the same MRI examination. All raw events and total counts were independently re-extracted from the original source tables for every study, with the source table and page recorded for each figure, specifically to obtain a harmonized ADDWOR-vs-ADDWR joint-effusion odds ratio for each study, avoiding both the mixed reference categories used in several of the original publications and reliance on non-representative sub-samples. For Liao et al. [26], effusion graded on a four-point scale was dichotomised as absent (grade 0) versus present (grades 1–3), consistent with the other studies reporting ordinal grades (ADDWOR 125/133; ADDWR 115/146; Table S6). The common-effect (Mantel–Haenszel) model showed an odds ratio (OR) of 1.72 (95% CI: 1.50–1.97; Z = 7.69; *p* < 0.0001), and the random-effects (DerSimonian–Laird) model showed an OR of 1.70 (95% CI: 1.42–2.03; Z = 5.85; *p* < 0.0001; 95% prediction interval: 1.12–2.57) (Figure 2). This represents an unadjusted joint-level association: joint effusion was more frequently observed in TMJs with anterior disc displacement without reduction than in those with reduction. Heterogeneity across studies was low to moderate (τ^2^ = 0.0300, I^2^ = 24.1%, 95% CI: 0.0–57.7%; Cochran’s Q = 21.08, df = 16, *p* = 0.175). A leave-one-out sensitivity analysis was performed, sequentially excluding each of the 17 studies (Figure S2). The pooled random-effects OR ranged from 1.65 to 1.79 and remained statistically significant after the exclusion of any single study; in particular, the exclusion of Koh et al. [33]—whose two internal disc-position classification tables were found to be mutually inconsistent (Table 1, footnote)—gave an OR of 1.79 (95% CI: 1.52–2.12), and the exclusion of the newly included study by Liao et al. [26] reproduced the previous estimate (OR = 1.65, 95% CI: 1.41–1.92). Visual inspection of the funnel plot (Figure S1) showed no marked asymmetry, and Egger’s regression test was non-significant (t = 0.07, df = 15, *p* = 0.94); however, with 17 studies the test has limited power, and publication or small-study effects cannot be excluded on this basis. Because 16 of the 17 included studies assessed TMJs bilaterally (i.e., non-independent, clustered observations from the same patient), an approximate design-effect sensitivity analysis was performed under assumed intraclass correlation coefficients (ICC = 0 to 0.8; Figure S3). Under these assumptions, the design-effect-corrected random-effects OR ranged from approximately 1.68 to 1.70, and its 95% confidence interval did not include 1 across the range examined. Because the true within-patient correlation is unknown and the method is an approximation, this analysis suggests that, under the assumed correlations, the association is unlikely to be explained by the non-independence of paired joints, but it cannot exclude this possibility.

## 4. Discussion

In this meta-analysis, joint effusion was more frequently observed in joints with ADDWOR than in joints with ADDWR (unadjusted joint-level random-effects OR: 1.70, 95% CI: 1.42–2.03). The pathogenesis of joint effusion may be related to three interrelated mechanisms: altered stress distribution, chronic inflammation and increased apoptosis [20,37,38]. According to previous evidence [18,20], joint effusion seems to be associated with intracapsular mechanical stress. Inflammation of the synovial membrane occurs due to excessive loading of the TMJ, resulting in stretching and distortion of the joint capsule, joint ligaments, and the surrounding tissues of the articular disc [39]. Pérez del Palomar et al. (2007) [37] demonstrated that in cases of anterior disc displacement, the distribution of stress within the disc changes significantly, showing increased compressive and tangential stress in the posterior band. They suggested that this altered stress distribution may be relevant to the transition towards anterior disc displacement without reduction (ADDWOR); however, the cross-sectional data pooled in the present review do not allow any such sequence to be assessed. According to Suenaga et al. [40], ADDWOR produces greater mechanical stress compared to ADDWR, as it involves more substantial elongation of the posterior attachment of the disc and of the articular capsule, contributing to the inflammatory response and the potential for effusion. Li et al. 2024 [27] also reported that disc displacement can lead to abnormal mechanical stress, with an increase in inflammatory mediator production and joint effusion. These biomechanical findings offer a possible, but untested, explanation for the results reported by Roh [18] and Thomas [19], who found a greater association of joint effusion with ADDWOR than with ADDWR. In particular, Thomas et al. [19] found a significant relationship between disc position and joint effusion, with a stronger association in patients with ADDWOR and disc perforation. Additionally, Roh et al. [18] reported a higher odds ratio of 4.61 (*p* < 0.001) for effusion in patients with ADDWOR compared to subjects with a normal disc position, consistent with the finding that joint effusion is more frequent in cases of displacement without reduction. Under mechanical stress, joint cells release proinflammatory molecules that activate a cascade of damaging events [41]. Increased mechanical stress activates an inflammatory response, characterized by the release of pro-inflammatory cytokines, including TNF-α and IL-1β, which disrupt the balance of the extracellular matrix and accelerate joint degeneration [42]; this condition promotes the release of other cytokines such as IL-6 and IL-8 [43,44], which play a central role in the inflammatory response [41]. Takahashi et al. [45] studied patients with internal dysfunction (ID) of the temporomandibular joint and identified several pro-inflammatory cytokines, including IL-1β, interleukin-6 (IL-6) and tumor necrosis factor alpha (TNF-α). Studies on synovial fluid have revealed the presence of prostaglandins E2 [46] in all inflamed temporomandibular joints, as well as metalloproteinases and collagenases, all of which determine an inflammatory state responsible for pain and swelling [45,47]. The production of free radicals and nitric oxide (NO), induced by mechanical stress and inflammation, increases programmed cell death (apoptosis) in the disc and synovial membrane [48,49,50] with a reduction in a tissue’s regeneration capacity and a consequent degeneration of joint structures [49,51]. Apoptosis, if not adequately balanced by cell proliferation, leads in fact to a reduction in the number of cells in the disc and synovium, contributing to the deterioration of joint structure. In addition, failure to dispose of apoptotic cells can amplify chronic inflammation by intensifying synovial fluid production and promoting intra-articular fluid accumulation [49,51]. All this involves a reciprocal maintenance mechanism in which chronic inflammation increases cell apoptosis and an inadequate metabolism of apoptotic cell breakdown products promotes tissue inflammation and joint degeneration; in this mechanism TNF-α plays an important role. TNF-α in fact promotes inflammation and induces apoptosis in synovial cells through the activation of death receptor-mediated pathways, contributing significantly to the pathogenetic mechanisms that result in joint damage [51]. This work has several limitations, primarily related to the type of study design. In all included studies, disc position and effusion were assessed on a single MRI examination, and most studies were based on retrospectively collected data, which may introduce selection bias. Selection bias could occur because, in clinical practice, MRIs are typically prescribed for patients with more severe symptoms, such as persistent pain or limited movement. As a result, patients without obvious symptoms or those not undergoing radiological exams could be excluded from the study, potentially leading to an overestimation of the association between disc displacement and joint effusion. Retrospective designs are susceptible to uncontrolled variables and data collection bias compared to prospective or randomized studies. Additionally, the cross-sectional nature of the comparison does not allow the temporal sequence between disc displacement and effusion, or a causal link, to be established; moreover, the pooled estimate is unadjusted and may be affected by confounding factors such as pain, degenerative joint disease, age and sex. Furthermore, a formal certainty-of-evidence assessment (e.g., GRADE) was not undertaken in this review; this should be considered when interpreting the overall strength of the evidence presented. A further limitation concerns the unit of analysis: in 16 of the 17 included studies, TMJs were assessed bilaterally, so that most joint-level observations are not statistically independent of one another, since two joints frequently originate from the same patient. To address this, a design-effect sensitivity analysis (ICC range 0–0.8) was performed (Section 3.5); the pooled odds ratio was similar across the assumed correlations, suggesting that, under these assumptions, clustering is unlikely to fully account for the observed association, although the true intraclass correlation for joint effusion is unknown and this limitation cannot be fully eliminated without patient-level data from the original studies. We recommend that future primary studies analyze bilateral MRI findings using methods that explicitly account for within-patient clustering (e.g., generalized estimating equations or mixed-effects models). Notably, one of the included studies, Khawaja et al. [28], already did so in its original analysis, using generalized estimating equations to account for within-participant correlation between the two TMJs; because the present meta-analysis relies on unadjusted joint-level 2 × 2 counts for all studies, this study-specific adjustment could not be carried forward into the pooled estimate, which is one of the reasons the design-effect sensitivity analysis above was performed. During preparation of this review, a complete re-audit of all study-level data extractions was also performed: raw 2 × 2 counts were re-verified against the original source tables for every included study, with the specific table and page recorded for each figure. This re-audit corrected a reference-category mismatch affecting some studies (e.g., Roh et al. [18]), replaced a non-representative outcome-balanced sub-sample previously used for Li et al. [27] with the full reported cohort, and identified an internal inconsistency between two tables of Koh et al. [33] (Table 1); a sensitivity analysis excluding Koh et al. [33] confirmed that this inconsistency does not materially affect the pooled result. Another aspect to consider is that, although all studies utilized MRI to assess disk displacement and joint effusion, there are differences in the criteria used to measure and define these outcomes. Further studies [18,20] have attempted to mitigate this issue by involving multiple radiologists or conducting repeated assessments; however, inter- and intra-operator variability remains a limitation. Subjective interpretation can lead to discrepancies in diagnosis, reducing the reliability of assessments, particularly in more ambiguous cases. Moreover, studies vary in their assessment of the severity of joint effusion, complicating direct results comparison. Some studies used ordinal grading scales with three or four grades [6,18,26,33,34], while others adopted binary criteria (presence or absence of effusion). This lack of standardization complicates direct comparisons of results across different studies and may lead to clinical discrepancies in patient management. The use of ordinal grading scales allows a more nuanced assessment of effusion than binary criteria [31,34]. Additionally, future research should investigate whether the type of disc displacement influences the severity of effusion. Comparing patients with ADDWR and ADDWOR over time could clarify whether ADDWOR is associated with more severe effusion. This would help determine if joint effusion is not only linked to mechanical stress but also to the specific type of disc displacement.

## 5. Conclusions

The results of our meta-analysis show a significant unadjusted joint-level association between joint effusion and anterior disc displacement, with a higher prevalence in joints with ADDWOR than in joints with ADDWR, based on 17 studies and 4254 TMJ-level observations.

- The pooled random-effects OR of 1.70 (95% CI: 1.42–2.03) indicates that joint effusion is more frequently observed in joints with ADDWOR than in joints with ADDWR; this unadjusted estimate was not materially altered by the omission of any single study or by an approximate design-effect sensitivity analysis for bilateral joints, although publication or small-study effects cannot be excluded.
- Joint effusion appears to be more frequently associated with ADDWOR than with ADDWR, possibly reflecting differing inflammatory and mechanical-stress patterns between the two forms of disc displacement; however, given the cross-sectional and retrospective nature of all included studies, this association should not be interpreted as evidence of a causal relationship.
- Further prospective studies are needed to clarify the underlying pathogenetic mechanisms and to better characterize the co-factors associated with joint effusion, ideally using analytic approaches that explicitly account for the non-independence of bilaterally assessed joints.

## Figures and Tables

**Table 1:** Characteristics of included studies.

Study	Countries	Design	Samples	Imaging	Inclusion Criteria	Exclusion Criteria	Results
Westesson and Brooks 1992 [5]	USA	Case–control	Tot: 390 TMJs in ADDWOR + ADDWR comparison: 437	-MRI 1.5T, 150 mm (Sagittal)	Patients presented with signs and symptoms of temporomandibular joint (TMJ) disorders and were referred for magnetic resonance imaging (MRI).	Patients who could not undergo MRI for any reason were not included.	MRI showed effusion in 7% of the joints with normal superior disk position, 40% of the joints with disk displacement with reduction, 50% of the joints with disk displacement without reduction.
Hosgor et al. 2019 [6]	Turkey	Retrospective cross-sectional	-Tot: 120 Male: 17 Female: 103 -Mean Age: 29.9 ± 12 years TMJs in ADDWOR + ADDWR comparison: 234	-MRI 0.5 T -FOV: 150 mm -T1, T2	-Unilateral, painful TMD -Pain-free contralateral TMJs evaluated as the control group.	-Patients with major deformities -Prior TMJ surgery -TMJ fractures -Known connective tissue or autoimmune diseases -Degenerative joint disease (e.g., osteoarthritis) -Condylar hypoplasia/hyperplasia/tumors -Concurrent use of steroids, narcotics, or muscle relaxants	Associations between marked effusion and both disc displacement and joint pain (*p* < 0.05).
Thomas et al. 2018 [19]	USA	Retrospective cohort study	-Tot: 47 Male/Female: Not specified in the original text -Mean age 45 years TMJs in ADDWOR + ADDWR comparison: 60	-MRI -T2, PD	-Pain primarily arthrogenous (from the joint) alongside coexisting myogenous (from the muscles) pain. -Underwent preoperative MRI of the TMJ. -Had initial non-surgical treatment.	Prior TMJ surgery. Primary neuropathic pain. Incomplete preoperative or postoperative outcomes of interest documented.	Associations between effusions and degenerative joint disease and higher probability of ADDWOR.
Manfredini et al. 2009 [20]	Italy	Cross-sectional	-Tot: 194 Male: 41 Female: 153 -Mean Age: 55.3 years TMJs in ADDWOR + ADDWR comparison: 223	RI 1.5T -T1, T2	Patients seeking treatment for TMD Patients who underwent a bilateral MRI of the TMJs during the diagnostic process. Patients who consented to use their MRI findings for scientific purposes.	-Patients with systemic diseases affecting the joint or masticatory muscles, such as: -Fibromyalgia -Other rheumatic diseases	Association between joint effusion ADDWOR (*p* = 0.008)
Ogura et al. 2019 [32]	Japan	Cross-sectional	Tot 49 Male: 13 Female: 36 Age: 46.3 years TMJs in ADDWOR + ADDWR comparison: 98	(1) MRI 1.5 T FOV: 130 mm × 130 mm	ADDWR and ADDWOR TMJ effusion Osteoarthrosis	Not reported	Logistic multivariate regression analysis Show that gender (odds ratio = 4.188, *p* = 0.012) and TMJ pain (odds ratio = 2.704, *p* = 0.027) were significant in patients with TMJ effusion.
Rudisch et al. 2001 [21]	Austria	retrospective cross-sectional	Tot: 41 Male: 9 Female: 32 Mean Age: 39.1 years TMJs in ADDWOR + ADDWR comparison: 52	-MRI 1.5 T, -FOV: 145 mm -T1, T2, PD	-Unilateral TMJ pain during palpation and mandibular opening. -Absence of TMJ-related TMD diagnosis of disk displacement.	-Patients with a history of trauma -Patients with myalgia, degenerative joint disease, collagen vascular disease	significant rela-tionship between the MR imaging diagnosis of TMJ ID and TMJ effusion (*p* = 0.000).
Li et al. 2024 [27]	China	Retrospective cross-sectional	Tot: 766 patients Male: 161 Female: 605 Mean Age: 31.68 ± 13.71 years TMJs in ADDWOR + ADDWR comparison: 1177	-MRI 1.5T-FOV: 140 mm × 140 mm -T1, T2, PDWI	-Patients with clinically diagnosed TMD.	-Patients with a history of trauma to the TMJ. -Patients with prior surgeries involving the TMJ. -Patients with systemic diseases that could affect TMJ function.	The odds of having: joint effusion were 1.740 higher in patients with ADDWR when compared to those with normal disc position were 1.726 higher in patients with joint sounds when compared to those without joint sounds., were 8.463 higher in patients with joint pain when compared to those without joint pain.
Roh et al. 2012 [18]	Korea	Retrospective cross-sectional	Tot: 254 Male: 92 Female: 162 Mean Age: 30.5 ± 12 years TMJs in ADDWOR + ADDWR comparison: 336	MRI 1.5 T	Patients with TMD	Patients with major deformities, TMJ fractures, or systemic diseases (e.g., rheumatoid arthritis).	ADDWR showed a 2.01 odds ratio (*p* < 0.01) of degenerative changes and a 2.85 odds ratio (*p* < 0.001) of JE. ADDWOR showed a 4.43 odds ratio (*p* < 0.001) of degenerative changes and a 4.61 odds ratio (*p* < 0.001) of joint effusions.
Khawaja et al. 2016 [28]	USA	retrospective cohort study	Tot: 158 Male: 64 Female: 94 Mean Age: 30.6 years TMJs in ADDWOR + ADDWR comparison: 136	(1) Hard tissue: CBCT (2) Soft tissue MR	-Consenting adult participants. -Presentation of symptoms related to TMD.	-Pregnant participants. -Participants with systemic rheumatological diseases, as determined by medical history. -Participants with DJD diagnosed via CT or CBCT scans.	ADDWR (OR = 2.5) was significantly associated with JE in the absence of DJD.
Koh et al. 2013 [33]	Korea	Cross-sectional	Tot: 253 TMD Male: 62 Female: 191 Age: 28.5 TMJs in ADDWOR + ADDWR comparison: 486	MRI 1.5 T -FOV: 140 mm × 140 mm. -T1, T2	-Diagnosis of Internal Derangement -MRI Imaging: Patients who underwent MRI examinations for the diagnosis of temporomandibular disorders (TMD) at the university hospital.	Not reported	There is no relationship between anterior disc displacement and joint effusion (*p* > 0.05). [Note: Table 3 of the original article (disc-position distribution) is internally inconsistent with Tables 6 and 7 of the original article (disc position × effusion cross-tabulation), which were used for data extraction in the present meta-analysis; see Discussion, Limitations. A sensitivity analysis excluding this study left the pooled estimate materially unchanged.]
Mizuhashi et al. 2022 [29]	Japan	Retrospective Cross-sectional study	Tot: 46 Male: 11 Female: 35 Age: 43.5 ± 16.6 years TMJs in ADDWOR + ADDWR comparison: 83	(1) MRI (1.5 T) (2) FOV: 130 mm × 130 mm (3) PD, T2	Patients diagnosed with ID through MRI.	Absence of ID	The presence of JE was larger on ADDWOR (χ2 = 7.47, *p* = 0.006) and the TMJ with pain (χ2= 7.50, *p* = 0.006)
Mizuhashi et al. 2024 [31]	Japan	Retrospective cross-sectional	Tot: 97 (patients not reported separately from TMJs; one TMJ per patient assumed, see Section 3.2) Male: 12 Female: 85 Age: 43.6 ± 19.3 years TMJs in ADDWOR + ADDWR comparison: 97	(1) MRI (1.5 T) (2) FOV: 130 mm × 130 mm (3) PD, T2	Patients diagnosed with TMD through MRI.	Absence of TMD	The results showed that the amount of synovial fluid in the joint effusion did not change with the existence of myofascial pain or TMJ pain.
Xu et al. 2023 [36]	China	Retrospective Case–control study	Tot 103 TMJs in ADDWOR + ADDWR comparison: 149	(1) MRI (3.0 T) (2) FOV: 100 mm × 100 mm (3) PD, T2, fast suppressed T2	Patients over 18 years of age. Patients who underwent MRI of the TMJ due to temporomandibular joint (TMJ) complaints.	Poor-quality images due to patient movement, which could negatively affect quantitative measurements. Patients with a history of trauma or surgical procedures affecting the TMJ region. Patients with lesions in the TMJ region. Patients with congenital anomalies affecting the TMJ.	MRI revealed effusion in 146 joints, including nine from Group CON. Group P had the highest median volume (66.65 mm^3^), while Groups CON (18.33 mm^3^) and NP (27.12 mm^3^) were similar. Effusion > 38.20 mm^3^ distinguished Group P from NP (AUC = 0.801, sensitivity 75%, specificity 78.9%). Larger effusion was linked to bone marrow edema, osteoarthritis, disc displacement, and higher retrodiscal tissue signal intensity (*p* < 0.05).
Yasan et al. 2023 [34]	Turkey	Retrospective Cross-sectional study	Tot: 162 Male: 27 Female: 135 Age: 37.63± 13.86 year TMJs in ADDWOR + ADDWR comparison: 288	(1) MRI (1.5 T) (2) FOV: 130 mm × 130 mm (3) PD, T2	Individuals presenting with at least one chronic symptom associated with TMD, such as TMJ pain, clicking or crepitus sounds, and limited mouth opening. Patients who have bilateral MRI scans available for analysis. Those with no prior history of treatment for TMJ before undergoing MRI.	Patients with a history of TMJ trauma, TMJ pathology, TMJ ankylosis, TMJ fractures, or rheumatological diseases.	In the ADDwoR group, the occurrence of convex and folded disc configurations, osteophyte formation, and grade 3 effusion was significantly elevated. This group also exhibited more pronounced clinical symptoms, along with higher degrees of disc deformation, osteophyte development, and severe joint effusion.
Yesiltepe et al. 2022 [30]	Turkey	Retrospective Cross-sectional study	Tot: 69 Male: 25 Female: 44 Age: 35.45 ± 14.16 years TMJs in ADDWOR + ADDWR comparison: 34	(1) MRI (1.5 T) (2) FOV: 130 mm × 130 mm (3) PD, T2	Patients who had at least one chronic symptom related to temporomandibular disorders (TMD), such as TMJ pain, clicking or crepitus sounds, and restriction in mouth opening. Patients who underwent bilateral MRI examinations for TMD. No previous TMJ treatment history prior to the MRI examination.	Patients with a history of TMJ trauma, TMJ pathology, TMJ ankylosis, TMJ fractures, or rheumatological diseases. Patients with any other condition that might interfere with MRI analysis or diagnosis.	Pathological changes in the LPM and TMJ can be visualized using MRI. In the R-DD group, higher rates of arthrosis at the condylar head, effusion, LPM area, and superior LPM intensity were observed.
Orlando et al. 2009 [35]	Italy	Retrospective Cross-sectional study	Tot: 77 Age: Not specified in the original text TMJs in ADDWOR + ADDWR comparison: 85	(1) MRI (1.5 T) (2) FOV: 15 cm (3) T1, T2	Patients referred from medical practitioners for the evaluation of TMJ disorders. MRI scans conducted for suspected TMJ effusion and/or disc displacement.	Patients with contraindications to MRI (e.g., metal implants, pacemakers, etc.). Insufficient quality of MRI images for proper assessment. Patients with other significant medical conditions that could interfere with TMJ diagnosis (e.g., systemic inflammatory diseases).	The analysis revealed a statistically significant correlation (*p* < 0.01) between disc displacement and joint effusion. The overall odds ratio for different types of disc displacement was 3.1, with anterior disc displacement without reduction showing the highest likelihood of MRI-detected effusion.
Liao et al. 2025 [26]	China	Retrospective cross-sectional	Tot: 154 patients (sex distribution not significantly different between groups). Mean age: ADDWR 27.7 ± 9.7, ADDWOR 26.6 ± 8.8 years. TMJs in ADDWOR + ADDWR comparison: 279 (ADDWR 146, ADDWOR 133)	MRI 1.5 T (FOV 120 mm × 120 mm); closed-and open-mouth oblique sagittal images; effusion graded on closed-mouth fat-suppressed T2	Patients attending the TMJ outpatient clinic (January 2022–June 2023) with TMD symptoms according to DC/TMD and ADD on MRI.	Maxillofacial trauma or tumours; previous TMJ surgery; congenital malformations; specific inflammation; poor image quality.	Effusion grade differed between ADDWR and ADDWOR (*p* < 0.05) and was an independent factor for ADDWOR in multivariable logistic regression (AUC 0.745).

**ADDwR** = Anterior Disc Displacement with Reduction; **ADDwoR** = Anterior Disc Displacement without Reduction; **ID** = Internal Derangement; **JE** = Joint Effusion; **MRI** = Magnetic Resonance Imaging; **FOV** = Field of View; **PD** = Proton Density; **TMJ** = Temporomandibular Joint; **TMD** = Temporomandibular Disorder; **DJD** = Degenerative Joint Disease; **CBCT** = Cone Beam Computed Tomography; **CT** = Computed Tomography.

## Data Availability

All the data are reported in the present manuscript.

## Supplementary Materials

The following supporting information can be downloaded at: https://www.mdpi.com/article/10.3390/dj14100663/s1, File S1: PRISMA 2020 Checklist; Table S1: Risk of bias assessment of cohort studies (Newcastle–Ottawa Scale); Table S2: Risk of bias assessment of cross-sectional studies (JBI Critical Appraisal Checklist); Table S3: Risk of bias assessment of case–control studies (JBI Critical Appraisal Checklist); Table S4: Studies excluded after full-text reading, with reasons; Table S5: Database-specific search strings; Table S6: Extracted joint-level 2 × 2 data for each included study; Figure S1: Funnel plot; Figure S2: Leave-one-out sensitivity analysis; Figure S3: Design-effect sensitivity analysis for bilateral joints.
