# Supplementary material for: TMJ Effusion and Anterior Disk Displacement: A Systematic Review and Meta-Analysis
Source: Dent J (Basel). 2026 Oct 12;14(10):663. doi: 10.3390/dj14100663 (PMC13703236; doi:10.3390/dj14100663)
Supplement: Supplementary file 1 [file dentistry-14-00663-s001.zip › Supplementary_Figures_Tables.pdf]

Supplementary Materials

TMJ Effusion and Anterior Disk Displacement: A Systematic Review and Meta-Analysis

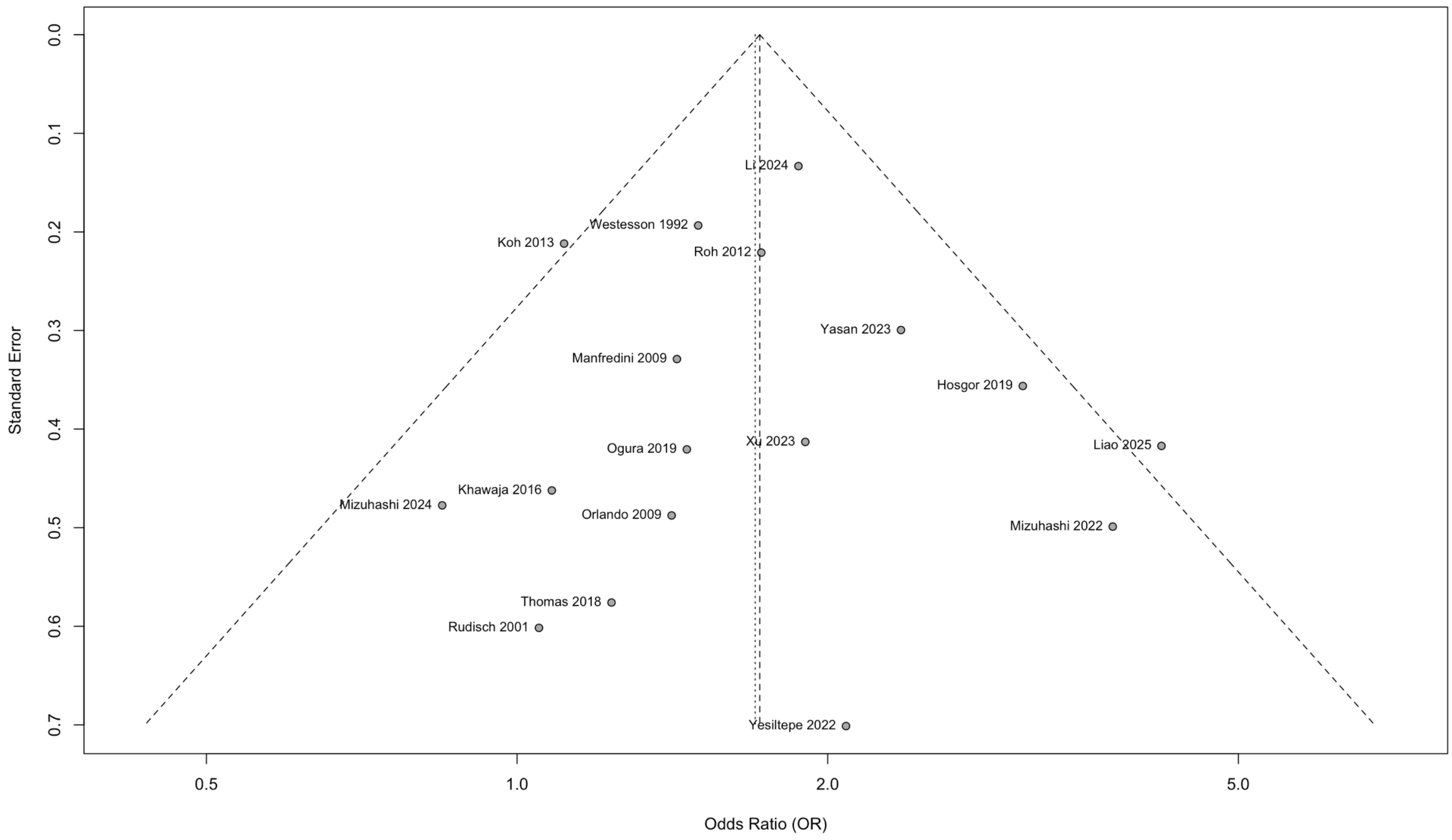

Figure S1. Funnel plot.

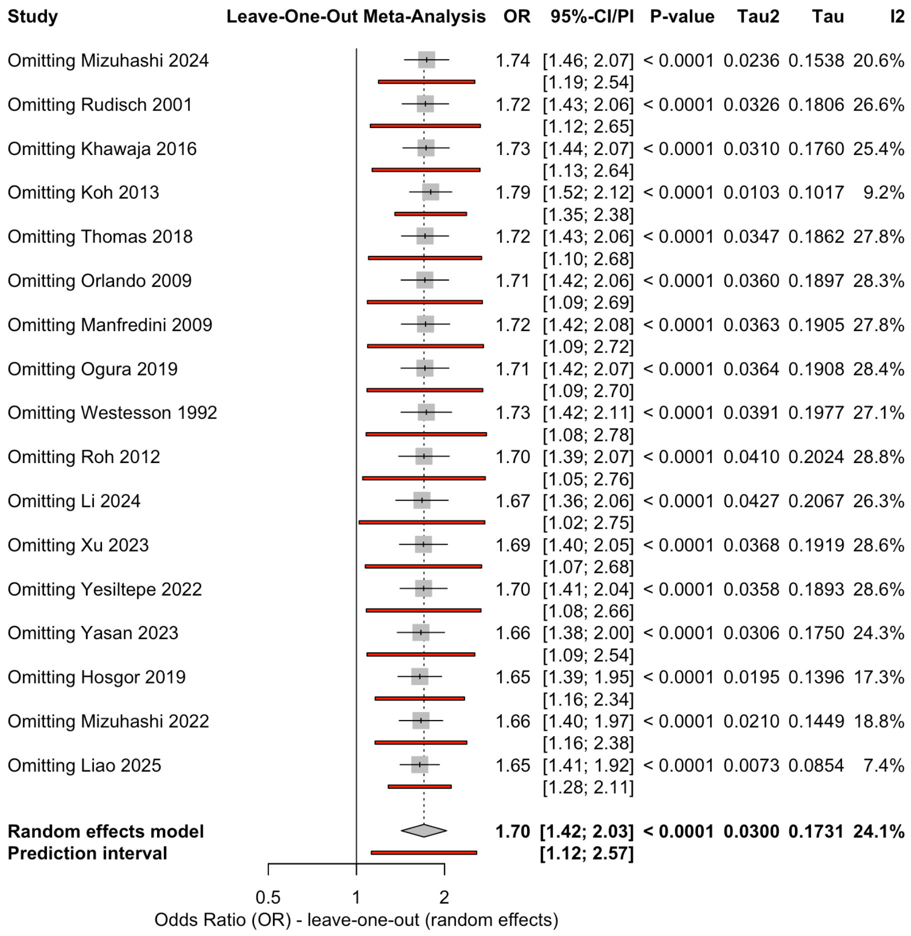

Figure S2. Leave-one-out sensitivity analysis.

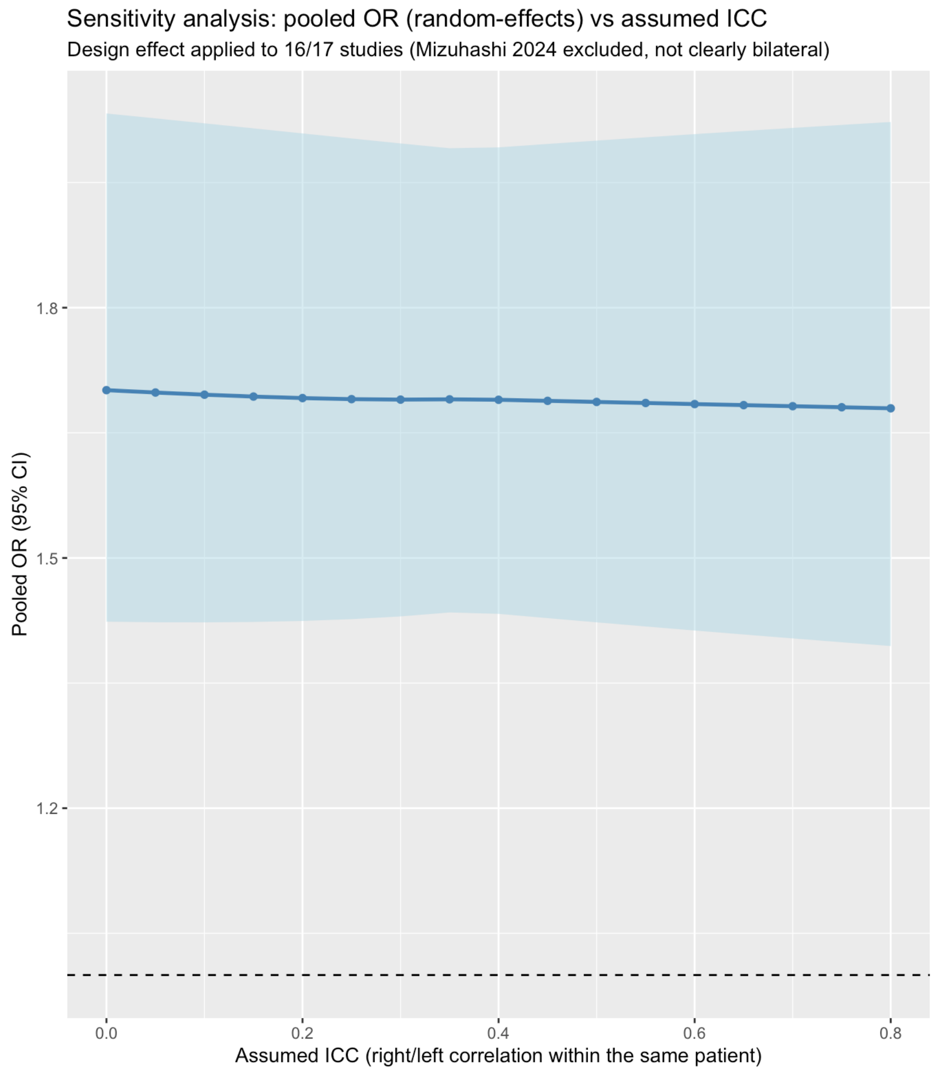

Figure S3. Sensitivity analysis: pooled OR (random-effects model) vs. assumed intraclass correlation coefficient (ICC).

Table S1. Quality assessment of included cohort studies (Newcastle–Ottawa Scale; NOS).

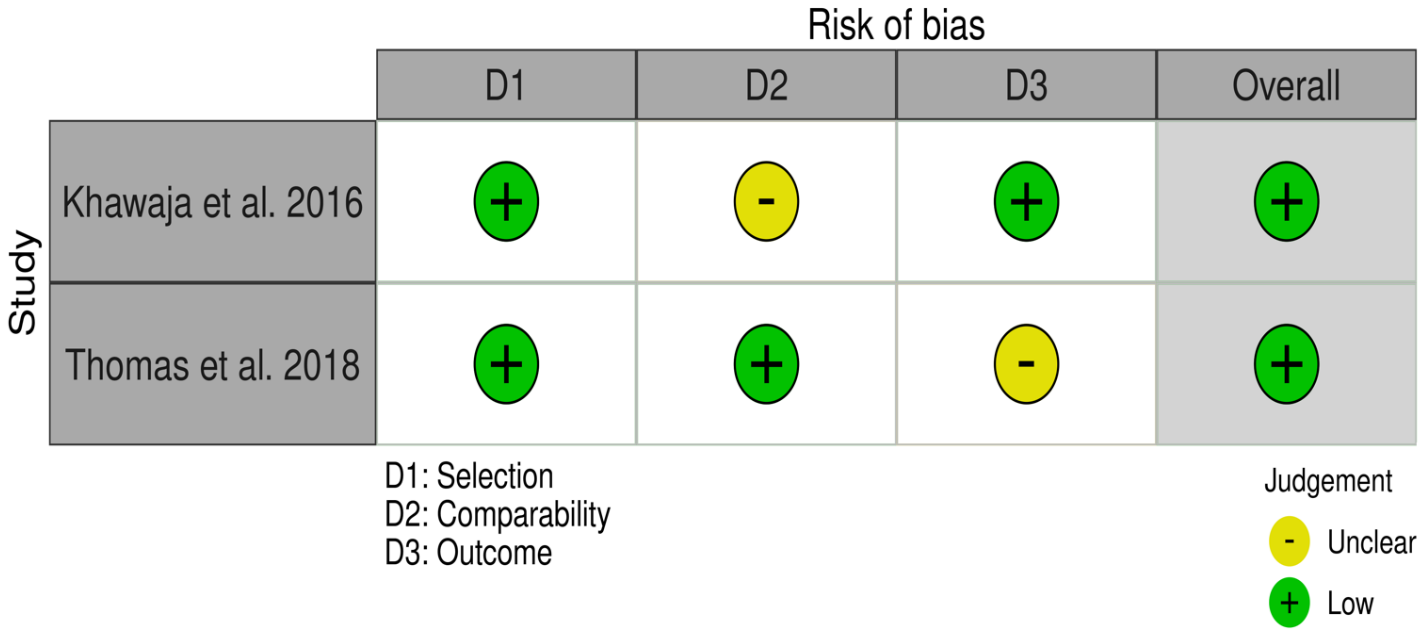

Overall judgment: low risk if no domain was rated high risk and no more than one domain was rated unclear; otherwise some concerns (shown as "Unclear"). See Section 2.5.

Table S2. Quality assessment of included analytical cross-sectional studies (Joanna Briggs Institute; JBI).

Risk of bias

Study	D1	D2	D3	D4	D5	D6	D7	D8	Overall
Hosgor et al. 2019	+	+	+	+	-	-	+	+	-
Koh et al. 2013	-	+	+	+	X	X	+	+	-
Li et al. 2024	+	+	-	+	+	+	-	+	-
Liao et al. 2025	+	+	+	+	+	+	+	+	+
Manfredini et al. 2009	+	+	+	+	X	-	+	+	-
Mizuhashi et al. 2022	+	+	+	+	-	+	+	+	+
Mizuhashi et al. 2024	+	-	+	+	-	X	+	+	-
Ogura et al. 2019	-	+	+	+	+	+	+	+	+
Orlando et al. 2009	-	+	+	+	X	X	+	+	-
Roh et al. 2012	+	+	+	+	+	+	+	+	+
Rudisch et al. 2001	+	+	+	+	-	X	+	+	-
Yasan et al. 2023	+	+	-	+	-	-	+	+	-
Yesiltepe et al. 2022	+	+	+	+	-	-	+	+	-

D1: Inclusion criteria clearly defined
D2: Subjects and setting described
D3: Exposure (disc position) measured validly and reliably
D4: Objective standard criteria for the condition
D5: Confounding factors identified
D6: Strategies to deal with confounding
D7: Outcome (joint effusion) measured validly and reliably
D8: Appropriate statistical analysis

Judgement
X High
- Unclear
+ Low

Overall judgment: low risk if no domain was rated high risk and no more than one domain was rated unclear; otherwise some concerns (shown as “Unclear”). See Section 2.5.

Table S3. Quality assessment of included case–control studies (Joanna Briggs Institute; JBI).

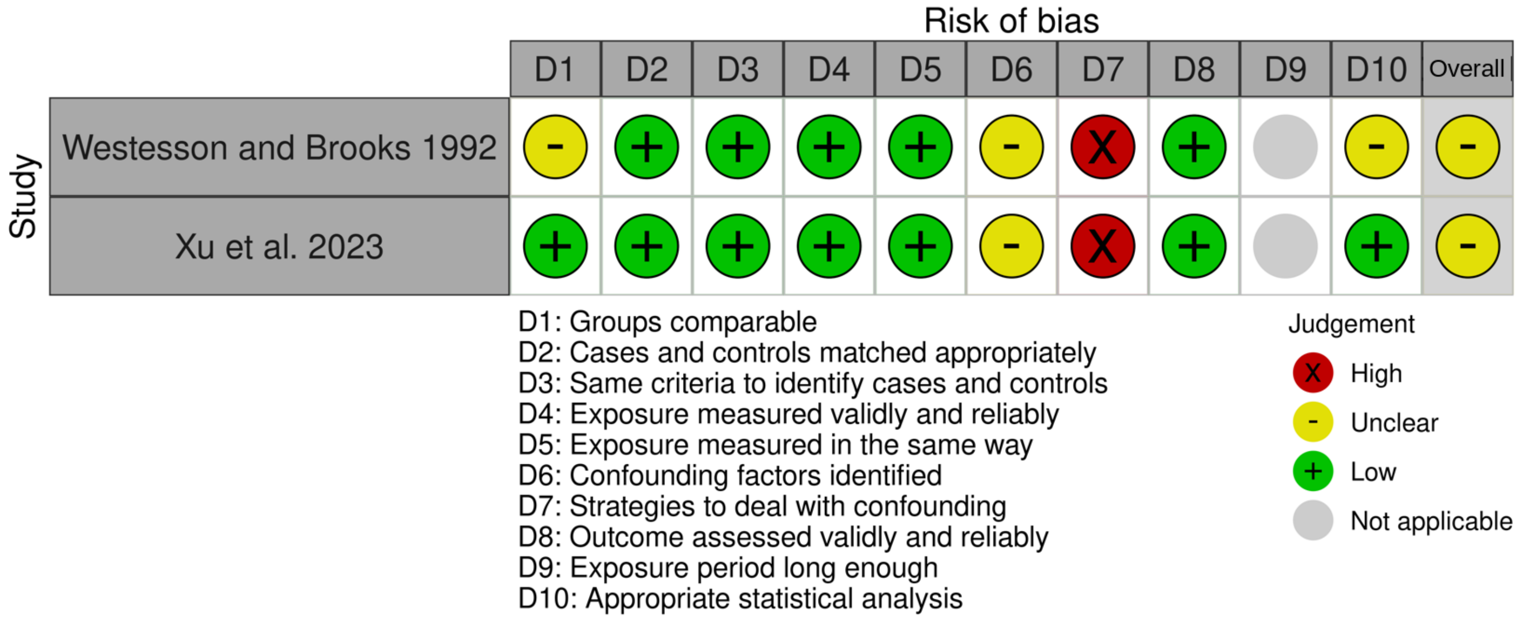

Overall judgment: low risk if no domain was rated high risk and no more than one domain was rated unclear; otherwise some concerns (shown as "Unclear"). See Section 2.5.

Table S4. Excluded studies with reasons.

#	Study (Author, Year)	Reason for exclusion
1	Jeon KJ, et al. 2021	Effusion analyzed as predictor, not as outcome
2	Suenaga S, et al. 2001	Outcome was nitric oxide/contrast enhancement, not effusion grading
3	Suenaga S, et al. 2016 [40]	Narrative review, no original data
4	Yano K, et al. 2004	Longitudinal pre/post-treatment design, not cohort comparison
5	Sano T, Westesson PL. 1995 [13]	Outcome was retrodiskal T2 signal, not joint effusion
6	Sang S, et al. 2024	Systematic review (secondary literature), not a primary study with original data
7	Wang S, et al. 2023	Outcome was lateral pterygoid muscle fat fraction/fibrosis, not joint effusion
8	Luo D, et al. 2023	Outcome was MRI texture analysis and fasciculation pattern of the lateral pterygoid muscle, not joint effusion
9	Shao B, et al. 2023	Outcome was condylar volume/joint-space morphology, not joint effusion
10	Azma R, et al. 2025	Outcome was automated (AI-based) disc-displacement classification accuracy in a pediatric population; joint effusion not assessed

Table S5. Database-specific search strings.

Database / Source	Search string	Fields searched / notes
MEDLINE (via PubMed)	(("temporomandibular joint"[tiab] OR TMJ[tiab]) AND "joint effusion"[tiab]) OR ("disk displacement"[tiab] AND "joint effusion"[tiab]) OR ("disk displacement"[tiab] AND ("temporomandibular joint"[tiab] OR TMJ[tiab])) OR ("joint effusion"[tiab] AND MRI[tiab]) OR ("disk displacement"[tiab] AND MRI[tiab])	Title/Abstract fields [tiab]; no MeSH restriction applied. Language: English. Species: humans. Date of last search: 14 September 2026
Scopus	TITLE-ABS-KEY(("joint effusion" AND TMJ) OR ("disk displacement" AND "joint effusion") OR ("disk displacement" AND TMJ) OR ("joint effusion" AND MRI) OR ("disk displacement" AND MRI))	Title, Abstract, Keywords (TITLE-ABS-KEY). Language: English. Species: humans. Date of last search: 14 September 2026
SciELO	("joint effusion" AND TMJ) OR ("disk displacement" AND "joint effusion") OR ("disk displacement" AND TMJ) OR ("joint effusion" AND MRI) OR ("disk displacement" AND MRI)	Advanced search, all indexed fields. Language: English. Species: humans. Date of last search: 14 September 2026
Virtual Health Library / LILACS (VHL/BVS)	(tw:("joint effusion" AND TMJ)) OR (tw:("disk displacement" AND "joint effusion")) OR (tw:("disk displacement" AND TMJ)) OR (tw:("joint effusion" AND MRI)) OR (tw:("disk displacement" AND MRI))	tw: = text word (title, abstract, subject descriptors). Language: English. Species: humans. Date of last search: 14 September 2026
Cochrane Library (CENTRAL — Trials and Reviews)	("joint effusion" AND TMJ):ti,ab,kw OR ("disk displacement" AND "joint effusion"):ti,ab,kw OR ("disk displacement" AND TMJ):ti,ab,kw OR ("joint effusion" AND MRI):ti,ab,kw OR ("disk displacement" AND MRI):ti,ab,kw	ti,ab,kw = Title, Abstract, Keyword fields. Language: English. Species: humans. Date of last search: 14 September 2026
ClinicalTrials.gov	Condition/disease: temporomandibular joint Other terms: ("joint effusion" OR "disk displacement") AND (TMJ OR MRI)	Basic search (Condition/Disease + Other terms fields). Date of last search: 14 September 2026
ICTRP (WHO Search Portal)	temporomandibular joint AND ("joint effusion" OR "disk displacement")	Basic search field. Date of last search: 14 September 2026

Table S6. Study-level data extraction table.

Study (Author, Year)	N patients	N TMJ	TMJ per patient	Events/Total ADDWOR | ADDWR (used in forest plot)	Effusion (event) definition	Source table/page	Population: complete or sub-sample	Ordinal → binary conversion
Westesson & Brooks 1992 [5]	390 (379 patients + 11 asymptomatic controls)	780 total; 437 used in the ADDWOR+ADDWR comparison (221+216)	2 (bilateral)	ADDWOR: 110/221 (50%) ADDWR: 86/216 (40%)	Binary: "no effusion" when no area of high signal, or only one line of high signal along the articular surface, was present on T2; "effusion" when more than one line of high signal was present.	Results section (percentages with denominator, not a dedicated table), p. 561 (AJR Am J Roentgenol 159:559-563).	Complete population; 780 TMJs in 4 categories (261 normal, 216 ADDWR, 221 ADDWOR, 82 arthrotic); the comparison uses only the 437 ADDWR+ADDWOR TMJs.	Not applicable (already binary).
Hosgor et al. 2019 [6]	120	240	2 (bilateral)	ADDWOR: 72/84 ADDWR: 99/150	3-grade ordinal scale: Grade 0 no effusion; Grade 1 moderate effusion (high-density linear signal on the articular surfaces); Grade 2 marked effusion (localized concentration in the inferior compartment).	Table 1, p. 941 (J Cranio-Maxillofac Surg 47:940-944).	Complete population (240 TMJ).	Binary = Grade 0 (absent) vs Grade 1+2 (present).
Thomas et al. 2018 [19]	47 (of 57 who underwent arthroscopy; only 47 with complete effusion + disc-position data)	94	2 (bilateral)	ADDWOR: 8/26 ADDWR: 9/34	Binary: "excess intra-articular synovial fluid", identified by hyperintensity in the joint space on T2 MRI (no grading scale).	Table 2, p. 98 (Br J Oral Maxillofac Surg 56:96-100).	Subset with complete data (47/57 patients).	Not applicable (already binary in the original article).
Manfredini et al. 2009 [20]	194	388	2 (bilateral)	ADDWOR: 35/133 ADDWR: 18/90	Binary: "no effusion" = no area or only faint lines of hyperintensity; "presence" = broad area of high signal intensity on T2.	Table I, p. 268 (Oral Surg Oral Med Oral Pathol Oral Radiol Endod 107:266-271).	Complete population (388 TMJ).	Not applicable (already binary).
Ogura et al. 2019 [32]	49	98	2 (bilateral)	ADDWOR: 18/37 ADDWR: 24/61	Binary, presence/absence based on T2 signal, criteria cited from an external reference (not detailed in the accessible text).	Table 1 (J Oral Maxillofac Radiol 7(2):30-33).	Complete population (98 TMJ).	Not applicable (already binary).
Rudisch et al. 2001 [21]	41	82	2 (bilateral)	ADDWOR (DDNR): 21/35 ADDWR (DDR): 10/17	Binary: high-signal-intensity area in the joint space; positive when more than one line of elevated signal is evident in ≥ 2 consecutive sections (Adame et al. criterion).	Table II (Oral Surg Oral Med Oral Pathol Oral Radiol Endod 92:566-571).	Complete population (82 TMJ).	Not applicable (already binary).

Study (Author, Year)	N patients	N TMJ	TMJ per patient	Events/Total ADDWOR | ADDWR (used in forest plot)	Effusion (event) definition	Source table/page	Population: complete or sub-sample	Ordinal → binary conversion
Li et al. 2024 [27]	766	1532	2 (bilateral)	ADDWOR: 291/743 ADDWR: 111/434	Binary: fluid signal extending over >4 consecutive slices on fat-suppressed T2, closed mouth.	Table 2 (complete cohort), p. 5 of 9 — NOT Table 3, a 458-vs-458 matched sub-sample used only for the original article's logistic regression (BMC Oral Health 24:314).	Complete population (1532 TMJ, Table 2) — correctly NOT the matched sub-sample in Table 3.	Not applicable (already binary).
Roh et al. 2012 [18]	254	508	2 (bilateral)	ADDWOR: 105/173 ADDWR: 77/163	4-grade ordinal scale: Grade 0 no signal; Grade 1 coating/dot; Grade 2 band; Grade 3 pooled collection.	Table 3, p. 285 (J Craniomaxillofac Surg 40:283-286).	Complete population (508 TMJ).	Binary = Grade 0 vs Grade 1-3.
Khawaja et al. 2016 [28]	158	312 total; 296 with complete sagittal classification used in the analysis	2 (bilateral; 4 TMJ missing)	ADDWOR (DDwoR): 9/25 ADDWR (DDwR): 38/111	Binary: high-intensity signal in the superior or inferior joint space on sagittal T2, closed mouth.	Table 2 (J Oral Maxillofac Surg 2016, online pagination).	296 of 312 total TMJs (sagittal-position data missing for 16 TMJ).	Not applicable (already binary).
Koh et al. 2013 [33]	253	506	2 (bilateral)	ADDWOR (DWOR): 236/336 ADDWR (DWR): 102/150	4-grade ordinal scale: Normal; E1 mild (dot or line of signal); E2 moderate (between mild and marked); E3 marked (pooled collection).	Tables 6 (right TMJ) + 7 (left TMJ) combined, p. 247-248 (Imaging Sci Dent 43:245-251).	Complete population (506 TMJ).	Binary = Normal vs E1+E2+E3.
Mizuhashi et al. 2022 [29]	46	83 (of 46×2=92 possible)	2 (bilateral; only TMJs meeting the "internal derangement" inclusion criterion were retained)	ADDWOR: 17/36 ADDWR: 9/47	Binary, presence/absence on T2 (no grading scale in this article).	Table 1 (J Oral Maxillofac Radiol 10(2):45-48).	Complete population meeting the inclusion criteria (83 TMJ).	Not applicable (already binary).
Mizuhashi et al. 2024 [31]	Not reported separately from TMJ count	97 (35 ADDWR + 62 ADDWOR)	Not reported; one joint per patient assumed (sex distribution 12 M + 85 F = 97 = number of TMJs).	ADDWOR: 44/62 ADDWR: 26/35	Presence of effusion in either joint compartment (Table 2). A separate 4-grade ordinal scale also exists (Table 3) but is NOT the source of the binary counts used here.	Table 2 (effusion region), not Table 3 (J Imaging 2024;10(10):241, open access).	Complete population (97 TMJ).	Binary = "no compartment" vs any region (superior/inferior/both).
Xu et al. 2023 [36]	103	206 (22 TMJ from 11 asymptomatic controls excluded, of 228 total); 149 used in the comparison (93 + 56)	2 (bilateral)	ADDWOR: 78/93 ADDWR: 41/56	Binary (presence/absence) based on fat-suppressed T2 signal.	Reconstructed from Table 2 (percentages for ADDWR/ADDWOR among TMJs with/without effusion, p. 1206), cross-checked with the group totals in Table 1.	Patients' TMJs only (healthy controls excluded).	Not applicable (already binary).
Yasan et al. 2023 [34]	162	324	2 (bilateral)	ADDWOR: 55/74 ADDWR: 118/214	4-grade ordinal scale: Grade 0 no strong T2 signal; Grade 1 dots/lines of bright T2 signal; Grade 2 bands of	Table 3 (J Craniomaxillofac Surg 51:441-447).	Complete population (324 TMJ).	Binary = Grade 0 vs Grade 1-3.

Study (Author, Year)	N patients	N TMJ	TMJ per patient	Events/Total ADDWOR ǀ ADDWR (used in forest plot)	Effusion (event) definition	Source table/page	Population: complete or sub-sample	Ordinal → binary conversion
					bright T2 signal; Grade 3 pooled bright T2 signal.			
Yesiltepe et al. 2022 [30]	69	126 (of 69×2=138 possible)	2 (bilateral; only TMJs meeting inclusion criteria were retained)	ADDWOR (R-DD): 10/18 ADDWR (R+DD): 6/16	Binary, presence/absence on fat-saturated FFE oblique sagittal T2 images.	Table 3 (J Stomatol Oral Maxillofac Surg 123:e973-e980).	Complete population (126 TMJ).	Not applicable (already binary).
Orlando et al. 2009 [35]	77	154	2 (bilateral)	ADDWOR (DDwoR): 18/27 ADDWR (DDwR): 34/58	Binary: "thin lines or areas of high signal intensity"; positive when more than one line of elevated signal is evident in ≥ 2 consecutive sections (same Adame criterion as Rudisch 2001).	Reported in the Results text, p. E189 (not in a stand-alone table) (Med Oral Patol Oral Cir Bucal 14:E188-E193).	Complete population (154 TMJ).	Not applicable (already binary).
Liao et al. 2025 [26]	154	279 (146 ADDWR + 133 ADDWOR)	Both joints included when available (≈1.8)	ADDWOR: 125/133 ADDWR: 115/146	4-grade ordinal scale on closed-mouth fat-suppressed T2: none; mild (point-like or linear high signal); moderate (between mild and marked); marked (pooling).	Table 2, p. 5 (BMC Med Imaging 2025;25:403).	Complete population (279 TMJ).	Binary = grade 0 (none) vs grades 1-3.

ADDWOR = anterior disc displacement without reduction; ADDWR = anterior disc displacement with reduction; TMJ = temporomandibular joint.
